# The protective effects of Pimavanserin against cerebral ischemia-induced brain injury

**DOI:** 10.1080/21655979.2021.1978617

**Published:** 2021-10-04

**Authors:** Xiang Li, Xiaoyan Tian

**Affiliations:** Department of General Practice, The Fourth Affiliated Hospital of Harbin Medical University, Harbin, HL, China

**Keywords:** Pimavanserin, cerebral ischemia stroke, blood-brain barrier, tight junction

## Abstract

The integrity of the blood-brain barrier (BBB) is mainly maintained by the brain vascular endothelial cells and the tight junctions amongst them. Pimavanserin is a novel agent approved for the treatment of Parkinson’s disease and exerts neuroprotective properties. The present study aims to explore the possibility that Pimavanserin might be an effective agent used for the treatment of cerebral ischemia stroke. Middle cerebral artery occlusion (MCAO) was established in mice, and oxygen-glucose deprivation/reoxygenation (OGD/R) was established in brain bEND.3 endothelial cells. Mice were randomly divided into four groups: (1) Sham operation group; (2). Pimavanserin (1 mg/kg); (3). MCAO; (4). Pimavanserin+ MCAO. We found that compared to the Sham group, the elevated neurological deficit score and brain water content increased production of inflammatory factors, increased BBB permeability, and downregulated Claudin 5 expression were observed in the MCAO group and were all dramatically reversed by the administration of Pimavanserin. Brain bEND.3 endothelial cells were treated with Pimavanserin before the exposure to OGD/R. The significantly increased lactate dehydrogenase (LDH) release, declined cell viability, increased endothelial permeability, downregulated Claudin 5 and Krüppel-like factors 6 (KLF6) were observed in the OGD/R group and were all reversed by the introduction of Pimavanserin. Lastly, the effects of Pimavanserin on the expression level of Claudin 5 and endothelial permeability in OGD/R-challenged endothelial cells were both abolished by the knockdown of KLF6. Taken together, our data revealed that Pimavanserin protected against cerebral ischemia injury by regulating the BBB integrity in a KLF6-dependent manner.

## Introduction

Stroke is a common disease with high mortality and disability rates [1,2]. According to the statistical data of the World Health Organization (WHO), as of the beginning of the 21st century, globally, an average of 15 million patients suffer from strokes annually, the majority of which are diagnosed with cerebral ischemia. Cerebral ischemic stroke is mainly induced by local hypoxia, and ischemia resulting from thrombus blockage of the cerebrovascular system. Currently, clinical therapy for ischemic stroke includes treatment with antithrombotic drugs to prevent thrombosis and dissolve existing thrombi, and neuroprotective agents to protect neurons against damage induced by ischemia/reperfusion. However, the limitations of these drugs are obvious. Treatment with anticoagulants reduces recurrent stroke but is accompanied by an increased risk of brain hemorrhage [3]. Moreover, deficiencies in neuroprotective treatment have been reported. Short half-lives, potential toxicity, and poor specificity of distribution are the main factors that limit the effect of neuroprotective agents on stroke [4]. For example, the half-life of Edaravone is only 5.4 minutes in the body, significantly reducing its effectiveness. Therefore, more effective therapies are still required for the treatment of ischemic stroke.

It is reported that after cerebral ischemic stroke, disruption of the blood-brain barrier (BBB) is the main inducer of death resulting from secondary brain injury [5]. The BBB consists of brain vascular endothelial cells, astrocytes end-feet, pericytes, and basement membrane [6], among which the brain vascular endothelial cells are its main component. Endothelial cells, together with the tight junctions, control the permeability of the BBB to protect the brain from neurotoxic substances and maintain cerebral homeostasis by managing the transmission of nutrition and trash between the brain tissue and the circulation [7]. Disruption of the BBB is regarded as an important pathological basis for the development of cerebral ischemic stroke [8]. Therefore, repair of the BBB might be an effective method for the treatment of cerebral ischemic stroke. Under a normal state, brain vascular endothelial cells maintain the exchange of substances between the brain and the blood by being selectively permeable to nontoxic materials. However, when brain vascular endothelial cells are damaged under the ischemic state, the permeability of blood capillaries is increased and the macromolecules in the blood freely enter the brain tissue, aggravating the severity of the brain injury induced by ischemia [9]. Besides, tight junctions among brain vascular endothelial cells are another important BBB component, mainly composed of intercellular transmembrane proteins, junction adhesion molecules (JAMs), and cytoplasmic attachment proteins [10,11]. Claudin, Occludin, and JAMs are reported to play critical roles in maintaining the regular function of tight junctions [12,13]. The BBB is disrupted by the increased permeability induced by the down-regulation of tight junction proteins, such as Claudin and Occludin, contributing to the development of cerebral ischemic stroke [14]. It is important to repair injured brain vascular endothelial cells and maintain the normal structure of tight junctions for the preservation of BBB integrity during cerebral ischemic stroke.

Pimavanserin is a selective serotonin inverse agonist first developed by Acadia Pharmaceuticals. It was approved by the US Food and Drug Administration (FDA) in April 2016 for the treatment of Parkinson’s Disease (PD). By selectively targeting the receptor of 5-HT_2A_, adverse effects arising from the activation of dopamine receptors have not been observed during the treatments with Pimavanserin [15]. Recently, protective effects of Pimavanserin on neurons have been reported [16]. However, it is unknown whether Pimavanserin has a beneficial effect against cerebral ischemia-induced brain injury.

The present study aims to investigate the pharmacological function of Pimavanserin in BBB integrity in an *in vivo* middle cerebral artery occlusion (MCAO) mouse model and an *in vitro* brain endothelial cells model. The underlying mechanism will be explored.

## Materials and methods

### *The establishment of the MCAO mice model and* in vivo *grouping*

Animal experiments were carried out in accordance with the protocols approved by the Institutional Animal Care and Use Committee of Harbin Medical University. Mice (7–9 weeks, 22–25 g) were purchased from HFK Bioscience Company (Beijing, China), then the environment was adjusted for 1 week. Animals were firstly anesthetized by administering with 50 mg/kg pentobarbital sodium (i.p.) and placed on a heating plate at 37°C, followed by making a midline incision expose the right common carotid artery (CCA), external carotid artery (ECA), and internal carotid artery (ICA). A surgical nylon monofilament (0.18 mm) was inserted from the right ECA into the ICA, and a heparin-dampened nylon suture was used to block CCA. An hour later, the monofilament was removed, and the clamp on the CCA was taken away, followed by reperfusion for 24 hours. Animals in the Sham group were treated with identical procedures without occlusion. For the *in vivo* experiments, mice were randomly divided into four groups: (1) Sham operation group; (2). Pimavanserin (1 mg/kg); (3). MCAO; (4). Pimavanserin (1 mg/kg) + MCAO. Animals were pretreated with Pimavanserin (1 mg/kg) before MCAO modeling.

### Neurological deficits

Six tests were involved in the neurological deficits: spontaneous activity, climbing, body proprioception, symmetry in limb movement, response to vibrissae touch, and symmetry of forelimb outstretching. The scoring was determined according to the standard described previously [17] with a 0 ~ 5point scale.

### Measurement of brain water content

The wet-dry method was utilized to evaluate the brain water content. In brief, the fresh brain tissues were isolated from each animal and weighed as the wet weight, followed by being dried in the oven. Subsequently, the dried brain tissues were weighed as the dry weight. Finally, the brain water content (%) was defined as (wet weight−dry weight)/wet weight×100%.

### Diffusion of sodium fluorescein assay

Na-fluorescein (Sigma, Missouri, USA) was administered intraperitoneally into the animals 1 h prior to the scarification, followed by being transcardially perfused with heparinized phosphate-buffered saline. Then, brain tissues and cardiac blood were collected and used for the fluorescent analysis. The uptake of Na-fluorescein was determined with a method described previously [18].

### Immunostaining

Brain tissues were isolated and fixed in the 4% paraformaldehyde, followed by being embedded with paraffin. A 7 μm section was made on the tissues, and the slides were incubated with the primary antibody against Claudin 5 (1:400, Invitrogen, California, USA) at 4°C for 24 h, followed by 3 washes and incubation with secondary antibody conjugated with Alexa Fluor 488 at room temperature for 1 hour. Lastly, the images were taken using an optical microscope (Leica, Wetzlar, German).

### Real-time PCR analysis

The brain bEND.3 endothelial cells were collected and used for the isolation of total RNAs with the TRIzol reagent (Invitrogen, California, USA). The extracted RNAs were transcribed into cDNAs with an iScript cDNA synthesis kit (Bio-Rad, California, USA), followed by performing the PCR REACTION WITH A SYBR Green Real-time PCR Master Mix (TOYOBO, Tokyo, Japan). Lastly, the 2^−ΔΔCt^ method was utilized to determine the relative expression level of target genes following the normalization with glyceraldehyde-3-phosphate dehydrogenase (GAPDH).

### ELISA assay

The release of monocyte chemoattractant protein-1 (MCP-1), interleukin (IL)-8, and IL-1β in the brain tissues were measured using the ELISA assay (Elabscience, Wuhan, China). In brief, cells were centrifugated at 300 × g for 5 min, and the supernatant was collected and then loaded into the 96-well plate before 1 h incubation at 37°C. Similar treatments were performed on the 5 gradient concentrations of the standard. Subsequently, the medium was removed, and wells were added with the conjugate reagents to be incubated at 37°C for 30 min, followed by adding the TMB solution for 15 min. Lastly, the stop solution was added to terminate the reaction and the microplate reader (Biotek, Vermont, USA) was used to measure the absorbance at 450 nm.

### Cell culture, OGD/R exposure, and lentivirus transduction

Brain bEND.3 endothelial cells were purchased from ATCC (Manassas, USA) and incubated in the DMEM with high glucose (4.5 mg/ml) containing 10% FBS at 37°C and 5% CO_2._ For the inducement of OGD/R condition, cells were deprived of glucose and oxygen using an anaerobic chamber (0% O_2_), followed by incubation for 3 h. Subsequently, cells were turned back to a normal incubator under the condition of 5% CO_2_/95% air for 24 h. The OGD/R was established.

Lentiviral-KLF6 and negative control shRNAs were from Sigma-Aldrich, USA. To produce lentiviral particles, we transfected the third-generation packaging plasmids (pVSV-G, pRSV-REV, and pMDLg/pRRE) and the lentiviral expression vector into 293 FT cells following the protocols as described before [19]. Lentiviral particles were collected and added to cells with 8 μg/ml polybrene for 24 hours.

### MTT assay

The cell viability of brain bEND.3 endothelial cells was measured by the MTT assay. Briefly, cells were mixed with 0.25 mg/ml MTT (Sigma-Aldrich, Missouri, USA) at 37°C for 3 h, and the medium was removed, followed by adding the dimethyl sulfoxide (DMSO) for the production of blue formazan. Lastly, the OD value at 630 nm was measured by the microplate reader (Mindray, Shenzhen, China) [20].

### LDH release

In brief, cells were seeded on a 96-well plate followed by being incubated at 37°C for 6 h. The LDH solution (Mlbio, Shanghai, China) was subsequently added into each well, and cells were incubated for 1 h at room temperature. Finally, the absorbance at 492 nm was measured using a microplate reader (Mindray, Shenzhen, China) for the calculation of LDH release.

### Western blot assay

The total proteins were extracted from cells and quantified with a BCA kit (Takara, Tokyo, Japan), followed by loading 30 μg proteins onto the SDS-PAGE. After separation, the proteins were transferred to the PVDF membrane (Millipore, Massachusetts, USA), followed by incubated with 5% skim milk [21]. Then, the membrane was incubated with the primary antibody against Claudin 5 (1:1500, Merck & Co Inc, New Jersey, USA), KL6 (1:2000, Merck & Co Inc, New Jersey, USA), and β-actin (1:10,000, Merck & Co Inc, New Jersey, USA), followed by being incubated with the secondary antibody (1:2000, Merck & Co Inc, New Jersey, USA) at room temperature for 1.5 hours. Finally, the ECL solution was used for the exposure, and the quantification of the bands was performed by the Image J software.

### Endothelial permeability was measured using FITC-dextran

Cells were seeded on the luminal side of filters (Corning, New York, USA), and 1 mg/mL FITC-dextran (Sigma, Missouri, USA) was added to the upper compartment, followed by incubation for 1 h. The absorbance of the lower chamber solution at 492 nm (excitation) and 520 nm (emission) wavelengths was measured using a microplate reader (Biotek, Vermont, USA) [5].

### Statistical analysis

Data obtained from the present study was expressed as Mean ± standard deviation (S.D.), and the GraphPad software was used for the data analysis. The Student’s t-test was used to analyze data between 2 groups, and the one-way ANOVA method was used for the analysis among groups. P < 0.05 was considered a significant difference.

## Results

Using an *in vivo* model, we investigated the effect of Pimavanserin on neurological deficits, expression of inflammatory factors, and BBB integrity in MCAO mice. Furthermore, we used an *in vitro* OGD/R- challenged brain endothelial cells model to investigate the potential benefits of Pimavanserin against OGD/R-induced cell death, aggravation of endothelial permeability, and the expression of the tight junction protein Claudin 5.

### Pimavanserin ameliorated neurological deficits in MCAO mice

**Figure 1.**
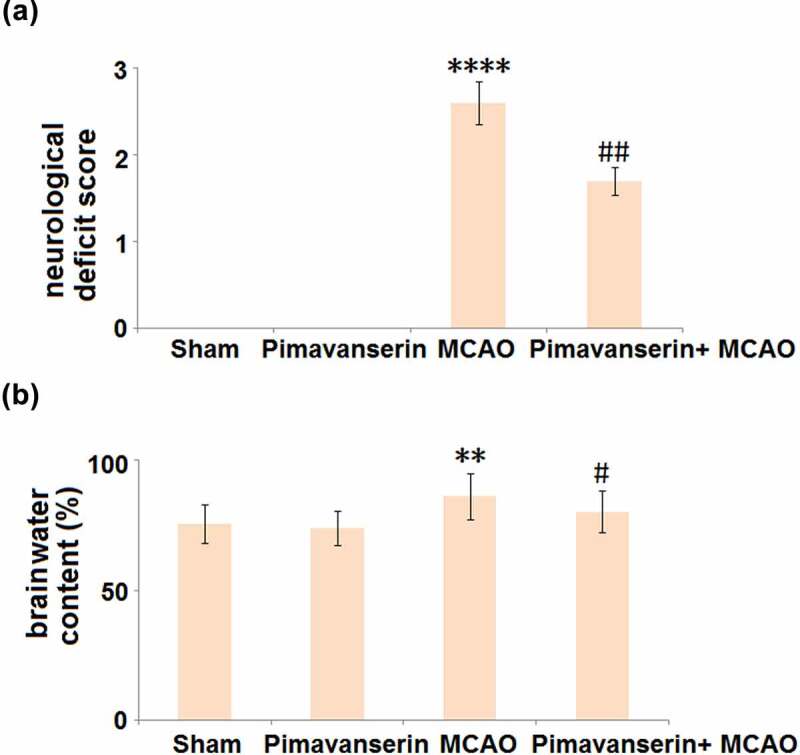
**Pimavanserin ameliorated neurological deficits in MCAO mice**. (a). Comparison of neurological deficit score in different groups; (b). Comparison of brain water content in different groups (**, ****, P < 0.01, 0.0001 vs. vehicle group; #, ##, P < 0.05, 0.01 vs. MCAO group)

The neurological deficit and brain water content were measured in each animal. As shown in Figure 1a, compared to the Sham group, no significant change in the neurological deficit score was observed in the Pimavanserin group. However, the neurological deficit score was significantly elevated in the MCAO group, after which it was dramatically suppressed by the treatment with Pimavanserin. In addition, compared to the Sham group, the brain water content (Figure 1b) was slightly changed from 75.6% to 73.8% in the Pimavanserin group and greatly promoted to 86.2% in the MCAO group, and then pronouncedly declined to 80.3% in the Pimavanserin+ MCAO group. These data indicate that the neurological deficits in MCAO mice were significantly alleviated by Pimavanserin.

### Pimavanserin inhibited the expressions of MCP-1, IL-8, and IL-1β in the brains of mice

**Figure 2.**
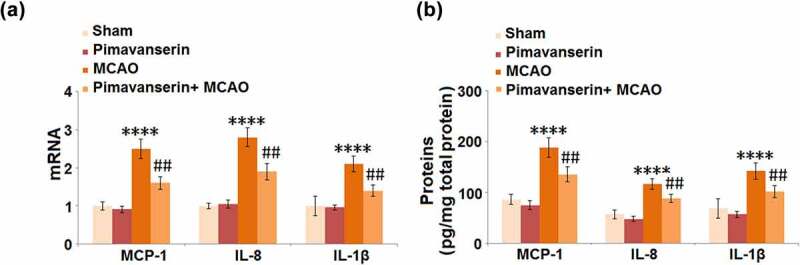
**Pimavanserin inhibited the expression of MCP-1, IL-8 and IL-1β in the brains of mice**. (a). mRNA of MCP-1, IL-8, and IL-1β; (b). Proteins of MCP-1, IL-8, and IL-1βas measured by ELISA (****, P < 0.0001 vs. vehicle group; ##, P < 0.01 vs. MCAO group)

The severe inflammation in brain tissue is an important pathological characteristic of ischemic injury. We further measured the production of inflammatory factors in the brain tissues of the animals. As shown in Figure 2a, compared to the Sham group, the mRNA expression levels of MCP-1, IL-8, and IL-1β were slightly changed in the Pimavanserin group and significantly decreased in the MCAO group, in which they were greatly elevated by the administration of Pimavanserin. In addition, compared to the Sham group, the production of MCP-1 was greatly elevated from 86.5 pg/mg total protein to 188.7 pg/mg total protein in the MCAO group, then dramatically declined to 135.7 pg/mg total protein by the treatment with Pimavanserin. The secretions of IL-8 in the Sham, Pimavanserin, MCAO, and MCAO+ Pimavanserin groups were 57.2, 48.3, 116.9, and 88.5 pg/mg total protein, respectively. Lastly, compared to the Sham group, the secretion of IL-1β was significantly promoted from 68.9 to 142.3 pg/mg total protein in the MCAO group but pronouncedly decreased to 102.1 pg/mg total protein in the Pimavanserin+ MCAO group. These data reveal that the severe inflammation in the brain tissues of MCAO mice was significantly suppressed by Pimavanserin.

### Pimavanserin preserved BBB integrity in MCAO mice by increasing the expression of Claudin 5

**Figure 3.**
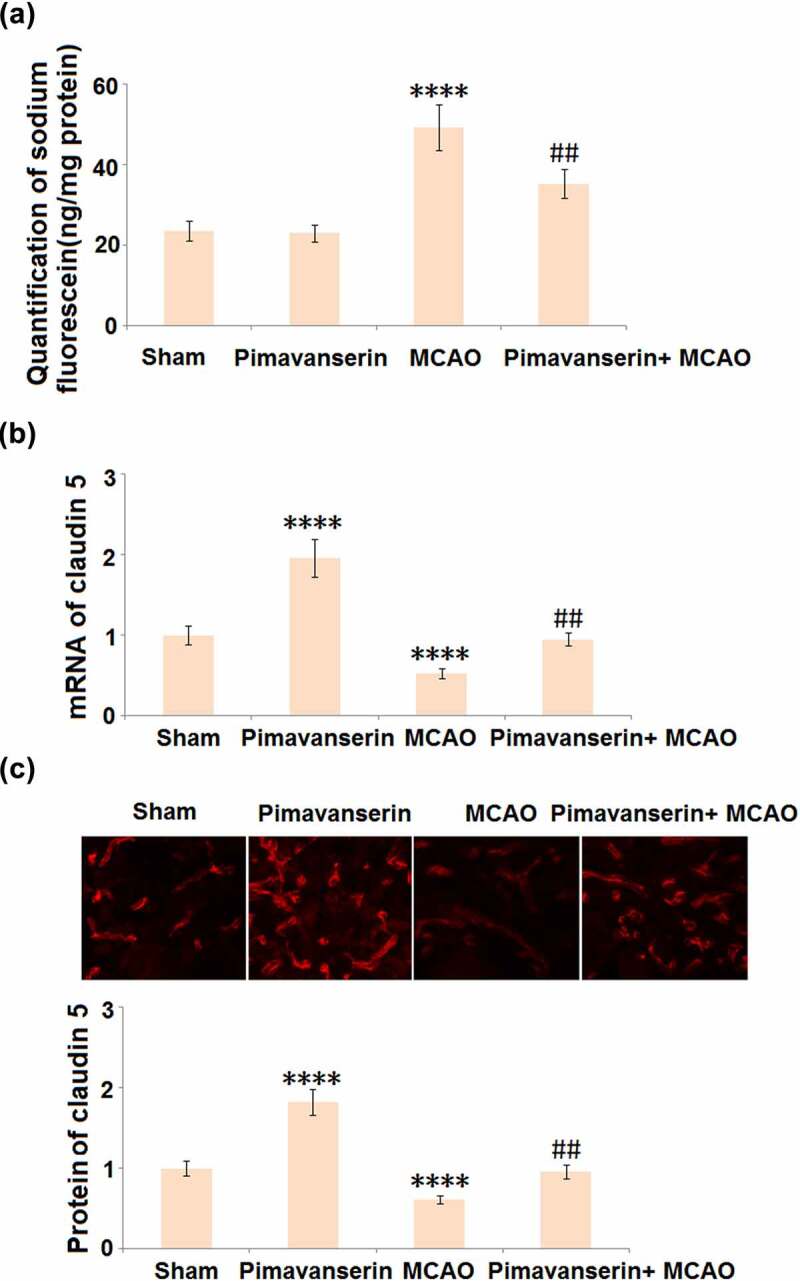
**Pimavanserin protected BBB integrity in MCAO mice by increasing the expression of claudin 5**. (a). BBB integrity was measured using diffusion of sodium fluorescein assay. (A). Quantification of sodium fluorescein diffusion in the brains of experimental mice; (b). mRNA of claudin 5; (c). Protein of Claudin 5 (****, P < 0.0001 vs. vehicle group; ##, P < 0.01 vs. MCAO group)

Compromised BBB integrity is an important pathological marker for cerebral ischemic stroke and contributes to secondary cerebral damages. The BBB integrity was evaluated after the MCAO modeling using the sodium fluorescein assay. As shown in Figure 3a, compared to the Sham group, the concentration of Na-fluorescein was slightly changed from 23.6 ng/mg protein to 23.1 ng/mg protein, then greatly elevated to 49.3 ng/mg protein in the MCAO group. It was, however, dramatically decreased to 35.3 ng/mg protein by the administration of Pimavanserin. Claudin 5 is a critical tight junction protein regulating the integrity of the BBB [22], the expression level of which in the brain tissues was further determined. As shown in Figure 3b-C, compared to the Sham group, Claudin 5 was significantly up-regulated in the Pimavanserin group and down-regulated in the MCAO group. Compared to the MCAO group, the expression level of Claudin 5 was dramatically elevated by the treatment of Pimavanserin. These data indicate that the disrupted BBB integrity in MCAO mice was significantly repaired by Pimavanserin.

### Pimavanserin protected cells against OGD/R- induced cell death

**Figure 4.**
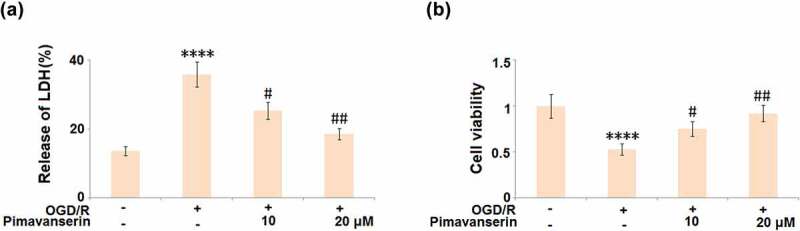
**Pimavanserin protected brain bEND.3 endothelial cells against OGD/R- induced cell death**. Cells were treated with 1, 2 μM Pimavanserin for 6 h, followed by exposure to oxygen-glucose deprivation (6 h)/reperfusion (24 h) (OGD/R). (a). Release of LDH;(b). Cell viability (****, P < 0.0001 vs. vehicle group; #, ##, P < 0.05, 0.01 vs. OGD/R group)

An oxygen-glucose deprivation/reperfusion (OGD/R) *in vitro* model was widely used to simulate the pathological symptoms of ischemia at the cellular level [23]. In the present study, brain bEND.3 endothelial cells were treated with 1 and 2 μM Pimavanserin for 6 hours before exposure to OGD/R. As shown in Figure 4a, compared to the control, the release of LDH was significantly elevated from 13.6% to 35.8% in the OGD/R group, then greatly declined to 25.3% and 18.6% by the introduction of 1 and 2 μM Pimavanserin, respectively. In addition, the cell viability (Figure 4b) was dramatically declined in the OGD/R group, but greatly promoted by the incubation with 1 and 2 μM Pimavanserin. These data reveal a protective property of Pimavanserin against OGD/R- induced cell death in brain endothelial cells.

### Pimavanserin attenuated OGD/R-induced aggravation of endothelial permeability

**Figure 5.**
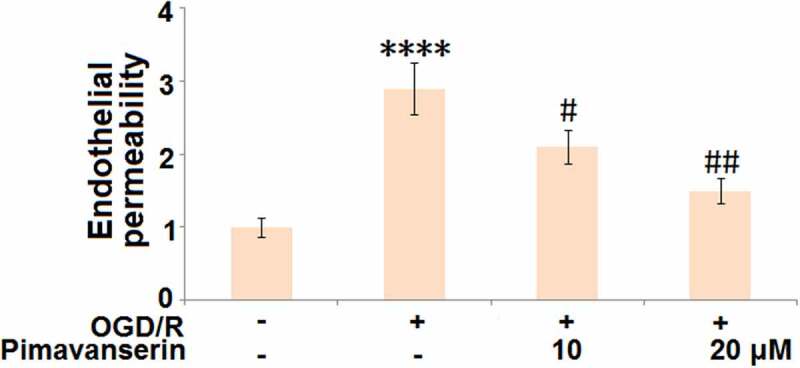
**Pimavanserin attenuated OGD/R- induced aggravation of endothelial permeability in brain bEND.3 endothelial cells**. Endothelial permeability was measured using FITC-dextran (****, P < 0.0001 vs. vehicle group; #, ##, P < 0.05, 0.01 vs. OGD/R group)

We further established an *in vitro* endothelial monocyte barrier model. As shown in Figure 5, compared to the control, the endothelial monocyte permeability was significantly increased in the OGD/R group, and then greatly decreased by the treatment with 1 and 2 μM Pimavanserin, indicating an inhibitory effect of Pimavanserin on the increased endothelial permeability induced by OGD/R.

### Pimavanserin restored the expression of Claudin 5 in OGD/R- challenged brain bEND.3 endothelial cells

**Figure 6.**
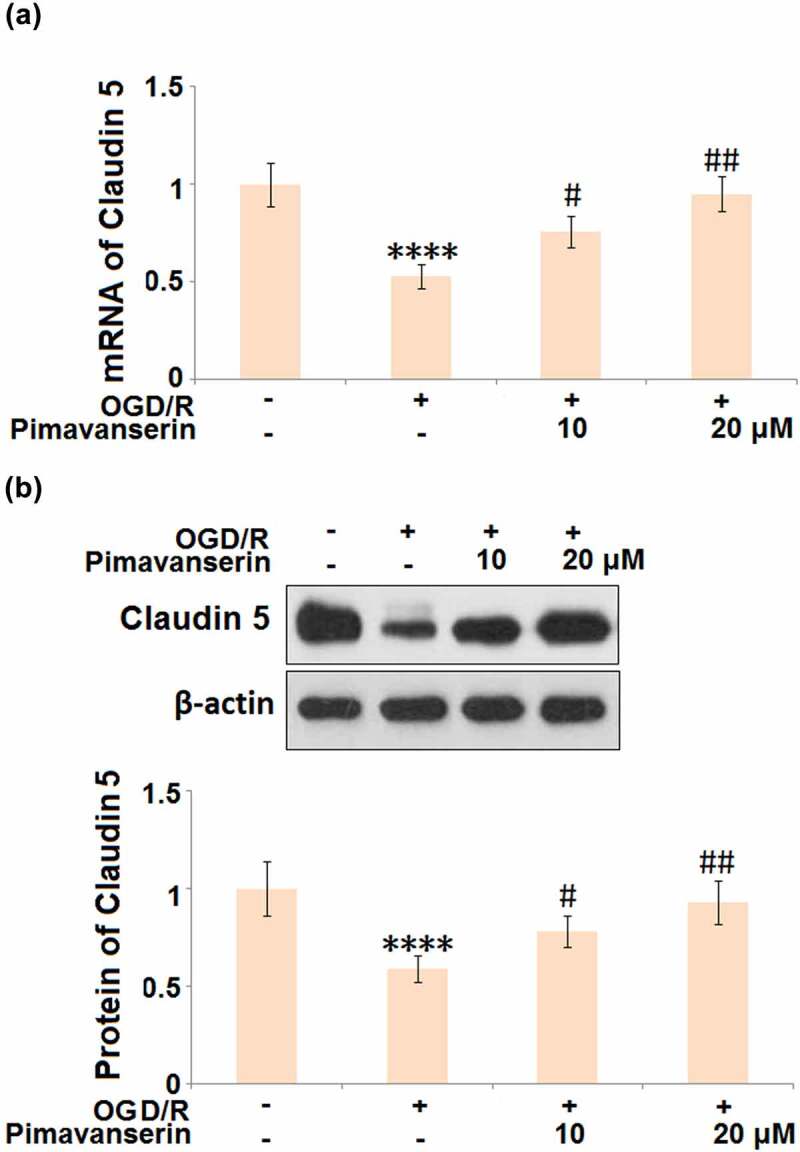
**Pimavanserin restored the expression of Claudin 5 in OGD/R- challenged brain bEND.3 endothelial cells**. (a). mRNA of Claudin 5; (b). Protein of Claudin (****, P < 0.0001 vs. vehicle group; #, ##, P < 0.05, 0.01 vs. OGD/R group)

Consistent with the *in vivo* experiment, the expression level of Claudin 5 was determined after cells were treated with 1 and 2 μM Pimavanserin for 6 h, followed by exposure to OGD/R. As shown in Figure 6, the expression level of Claudin 5 was dramatically declined in the OGD/R group, which was significantly reversed by the administration of 1 and 2 μM Pimavanserin, indicating that the down-regulated Claudin 5 induced by OGD/R was restored by Pimavanserin.

### The protective effects of Pimavanserin against OGD/R- induced endothelial permeability are mediated by KLF6

**Figure 7.**
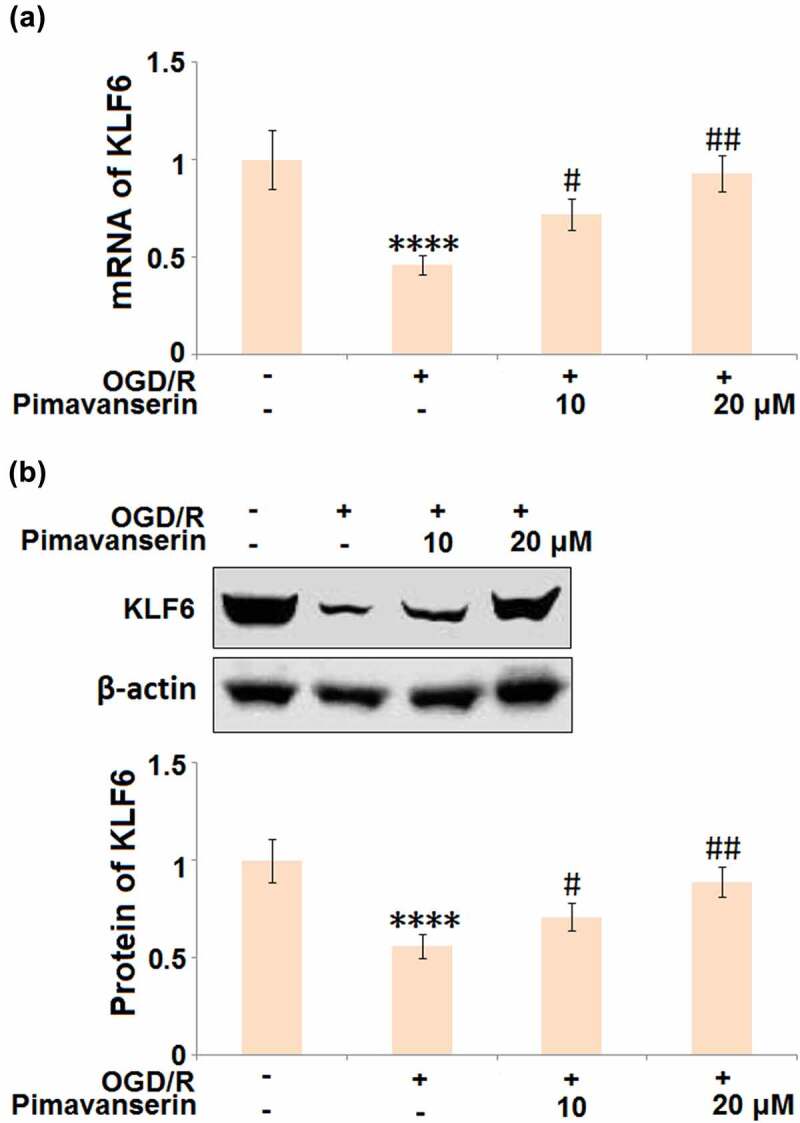
**Pimavanserin prevented OGD/R- induced reduction of KLF6 in brain bEND.3 endothelial cells**. Cells were treated with 1, 2 μM Pimavanserin for 6 h, followed by exposure to oxygen-glucose deprivation (6 h)/reperfusion (24 h) (OGD/R). (a). mRNA of KLF6; (b). Protein of KLF6 (****, P < 0.0001 vs. vehicle group; #, ##, P < 0.05, 0.01 vs. OGD/R group)

KLF6 is an important positive transcriptional factor that regulates the expression levels of tight junction proteins, such as ZO-1, Occludin, and Claudin-5 [24]. As shown in Figure 7, we found that the expression level of KLF6 was significantly suppressed in the OGD/R group, then greatly elevated by the introduction of 1 and 2 μM Pimavanserin.

**Figure 8.**
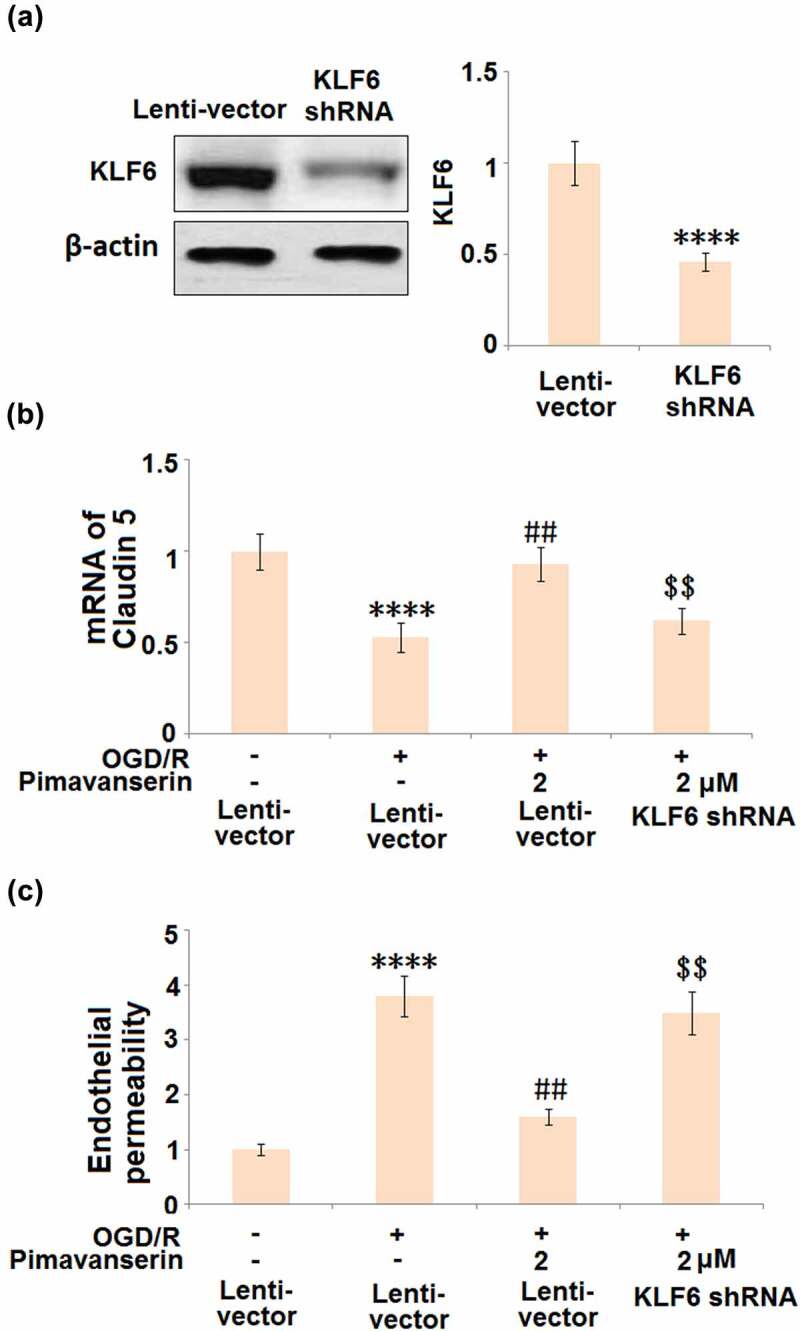
**Knockdown of KLF6 abolished the protective effects of Pimavanserin against** OGD/R- induced endothelial permeability. Cells were transduced with lentiviral KLF6, followed by stimulation with OGD/R with or without 2 μM Pimavanserin. (a). Western blot analysis revealed successful knockdown of KL6; (b). mRNA of Claudin 5; (c). Endothelial permeability was measured using FITC-dextran (****, P < 0.0001 vs. vehicle group; ##, P < 0.01 vs. OGD/R group; $$, P < 0.01 vs. OGD/R+ Pimavanserin)

To verify whether the protective effects of Pimavanserin against OGD/R- treated endothelial cells were mediated by the upregulation of KLF6, cells were transduced with shRNA-KLF6, followed by stimulation with OGD/R with or without 2 μM Pimavanserin. As shown in Figure 8a, compared to the control, the expression level of KLF6 was significantly declined by the transfection of shRNA-KLF6, indicating a successful establishment of KLF6-knockdown endothelial cells. The declined expression level of Claudin-5 (Figure 8b) in the OGD/R group was significantly elevated by the treatment with Pimavanserin, which was dramatically reversed by the knockdown of KLF6. In addition, as shown in Figure 8c, the increased permeability in the OGD/R group was significantly suppressed by the introduction of Pimavanserin, which was greatly reversed by the knockdown of KLF6. These data verify that the protective effect of Pimavanserin on OGD/R- treated endothelial cells is mediated by KLF6.

## Discussion

In this study, we aimed to explore the possibility that Pimavanserin might be an effective agent used for the treatment of cerebral ischemia stroke. Using a MCAO mouse model, we found that Pimavanserin ameliorated neurological deficits, inhibited the expressions of inflammatory factors including MCP-1, IL-8, and IL-1β, and preserved BBB integrity by increasing the expression of Claudin 5. Furthermore, in the OGD/R- challenged cells model, Pimavanserin significantly mitigated OGD/R-induced cell death, and prevented aggravation of endothelial permeability. Importantly, we found that the protective effects of Pimavanserin in OGD/R- challenged cells were mediated by KLF-6.

The BBB is an endothelial barrier consisting of brain vascular endothelial cells, pericytes, astrocytes, and a base membrane. It is located between the brain tissue and blood circulation. Along with the surrounding neurons and extracellular matrix, the BBB constitutes the neurovascular unit (NVU) to maintain the homeostasis of the microenvironment in the central nervous system (CNS) [25]. The CNS is separated from the circulatory and immune systems by the BBB, preventing the CNS from encountering neurotoxins and neurotransmitters. The substances in the circulation are strictly screened by the BBB, with the nutrition transferred from the blood to the brain tissues and metabolites cleared from the brain tissues into circulation [26]. Disruption of the BBB is a classic characteristic of cerebral ischemia stroke and contributes to secondary cerebral injury [27]. In the present study, we established a MCAO model in mice to simulate the pathological symptoms of cerebral ischemia stroke according to previous reports [28,29], verified by the increased neurological deficit score, brain water content, and excessive production of inflammatory factors. After the treatment with Pimavanserin, the neurological deficits in MCAO mice and the severe inflammation in the brain tissue were significantly ameliorated, indicating a promising protective effect of Pimavanserin against the pathological changes in MCAO mice. In addition, the increased BBB permeability in MCAO mice was found to be significantly reversed by the treatment with Pimavanserin. The *in vitro* experiments showed that the injured endothelial cells and increased endothelial monocyte permeability in OGD/R- challenged brain vascular endothelial cells were significantly alleviated by Pimavanserin. The results from the *in vivo* and *in vitro* assays revealed that the protective effects of Pimavanserin might be associated with its therapeutic impact on the disrupted BBB.

The junctional complexes among endothelial cells are important structures determining the integrity and are mainly composed of adherents junctions (AJ) [30], tight junctions (TJ) [12], and gap junctions (GJ) [31]. TJs span the apex of the intercellular barrier of epithelium or endothelial tissue, regulating the distance between the adjacent endothelial cells and maintaining the permeability of the vascular endothelial cell bypass [25]. TJs are composed of Occludins, Claudins, and junctional adhesion molecules (JAMs), and they bind to the actins to form a barrier [32]. The expression level of Claudin-5 in brain tissue is reported to be significantly higher than other Claudins [33]. Claudin-5 is a transmembrane protein consisting of 4 transmembrane domains, 2 extracellular loops, 1 intracellular loop, 1 amino terminal, and 1 carboxyl terminal [34]. It is reported that in the Claudin-5 knockout mice, no significant disruption is observed in TJs. However, the permeability of the BBB to molecules smaller than 800 Da is dramatically increased [21], indicating that Claudin-5 impacts barrier function by regulating the permeability of the BBB. In the present study, both in MCAO mice and OGD/R- treated brain vascular endothelial cells, the declined expression level of Claudin-5 was significantly elevated by the introduction of Pimavanserin, implying that Claudin-5 might be involved in the mechanism of the protective effect of Pimavanserin. KLF6 is an important transcriptional factor recently found to be involved in the activation of the transcription of multiple tight junction proteins, including Claudin-5 [24,35]. We found that the expression level of KLF6 was significantly suppressed in the OGD/R- treated brain vascular endothelial cells, indicating that KLF6 might be involved in the pathological mechanism underlying BBB disruption post-ischemia. After the treatment with Pimavanserin, KLF6 was significantly up-regulated. In addition, the effects of Pimavanserin on the expression level of Claudin 5 and endothelial permeability were both abolished by the knockdown of KLF6, indicating that the protective effect of Pimavanserin in cerebral ischemia injury is mediated by KLF6. In our future work, the binding appetency between Pimavanserin and KLF6 will be investigated to further understand the molecular functional mechanism of Pimavanserin.

The pathophysiological mechanism of cerebral ischemia is complicated. A variety of risk factors have been involved in the pathogenesis of stroke, such as lipids, pro-inflammatory cytokines, and neurotransmitters. Serotonin has been reported to play a wide range of roles in regulating brain diseases, including Alzheimer’s disease, brain trauma injury, and Parkinson’s disease [36]. Interestingly, platelet-derived Serotonin participates in regulating the physiological processes of thrombosis and homeostasis [37], which are the key steps of stroke. Therefore, we speculate that the protective effects of Pimavanserin in MCAO mice might be associated with its physiological function in Serotonin. Consistently, administration of serotonin selective reuptake inhibitors (SSRIs) has been shown to improve stroke recovery in non-depressed patients when given for 3 months after the stroke, with highly favorable safety conditions and a favorable benefit/risk ratio [38]. However, the enigma is still to be elucidated. Therefore, further investigations are warranted to clarify the underlying mechanism.

Here, we also acknowledge several potential limitations of our work. In the *in vivo* experiments, we tested for the effect of Pimavanserin on several inflammatory factors such as MCP-1, IL-8, and IL-1β. We found that Pimavanserin inhibited the expression of MCP-1, IL-8, and IL-1β at both mRNA and protein levels, indicating the potential anti-inflammatory benefits of Pimavanserin in the treatment of cerebral ischemia stroke. It has been well known that neuroinflammation plays a critical role in BBB damage and the underlying mechanisms are complicated. However, in this study, the effect of Pimavanserin on neuroinflammation is not clarified exhaustively. Additionally, damage and dysfunction in various types of cells, including neurons, microglia, and astrocytes, have been implicated in the pathogenesis of cerebral ischemia. In this study, we only focused on the pharmacological function of Pimavanserin in brain endothelial cells. Therefore, future studies will provide more evidence on how Pimavanserin prevents neuroinflammation and neurological deficits in ischemic stroke and provide us with a more complete picture.

## Conclusion

In conclusion, our data revealed that Pimavanserin protected against cerebral ischemia injury by maintaining BBB integrity in a KLF6-dependent manner.
